# Sleep bruxism in children is associated with multiple dimensions of anxiety

**DOI:** 10.4317/jced.64478

**Published:** 2026-09-15

**Authors:** Pablo Rivadeneira, Roberto Bellsham, Matías Miranda, Valeria Ávila, Diego De Nordenflycht

**Affiliations:** 1 Faculty of Dentistry, Universidad Andrés Bello, Viña del Mar, Chile; 2 Private practice, Viña del Mar, Chile

**Keywords:** Anxiety, bruxism, child, psychometrics.

## Abstract

**Background:**

To evaluate overall and domain-specific anxiety in children with and without sleep bruxism using the multidimensional Spence Children’s Anxiety Scale - Parent Version (SCAS-P).

**Materials and Methods:**

A comparative cross-sectional study was conducted in pediatric patients aged 7-12 years attending a university dental clinic. Sleep bruxism was assessed according to the ICSD-3 criteria. The Spence Children’s Anxiety Scale - Parent Version (SCAS-P), considering both the total score and individual subscales, was applied to all subjects for a multidimensional assessment of anxiety. Group comparisons between children with sleep bruxism (SB) and without sleep bruxism (No-SB) were performed using the Mann-Whitney U test at p=0.05.

**Results:**

Of the 122 children who met the inclusion criteria and were included in the analysis, 88 were classified as having SB. Children with SB showed significantly higher total anxiety scores compared with children without SB (p=0.002). Higher scores were also observed in children with SB for panic/agoraphobia, separation anxiety, physical injury fears, and social phobia (all p<0.05), with small-to-moderate effect sizes.

**Conclusions:**

Sleep bruxism in children is associated with higher anxiety levels, particularly in specific anxiety domains. These findings highlight the relevance of psychosocial assessment in pediatric patients.

## Introduction

Bruxism has long been a subject of considerable interest in dental and medical literature because of its complex nature and associations with medical and behavioral factors. According to the most recent international consensus, bruxism is defined as repetitive masticatory muscle activity characterized by clenching or grinding of the teeth and/or bracing or thrusting of the mandible [1]. This definition emphasizes that bruxism is a motor behavior rather than a movement or sleep disorder in otherwise healthy individuals, and it presents as two distinct circadian forms during wakefulness (awake bruxism) and during sleep (sleep bruxism) [1,2]. In pediatric populations, sleep bruxism may produce clinical consequences, including tooth wear, headaches, and temporomandibular disorders [3,4]. The prevalence of sleep bruxism in children is reported with considerable variability in the literature, largely due to differences in diagnostic methods and the age groups studied [5]. Clinical studies show that prevalence rates vary from 3.5% to more than 40%, with some systematic reviews reporting peaks up to 50%, with no notable differences between sexes. Despite this variability, the general trend shows that sleep bruxism is more frequent in younger children and then declines as they approach adolescence and adulthood [2].

Current evidence suggests that bruxism is regulated by the central nervous system, although it can be influenced by peripheral stimuli [2], and that its etiology involves a complex relationship of biological, psychological, and morphological factors [6,7]. Within these etiological factors, psychosocial and affective factors have frequently been associated with sleep bruxism in children [3,8-10]. Studies have shown that children with a higher frequency of sleep bruxism episodes often exhibit specific traits, such as high levels of neuroticism (characterized by anxiety, anger, and irritability) as well as a strong sense of responsibility and self-discipline [8].

The relationship between bruxism and anxiety is one of the most frequently investigated associations [3,6,11]. Some studies have reported associations between anxiety and teeth clenching/grinding behavior in children [6,9,10]. Several studies have reported higher anxiety scores in children with sleep bruxism than in those without the condition [5,6,9,10]. Importantly, multidimensional assessment using the Screen for Child Anxiety and Related Disorders (SCARED) has shown higher total anxiety, separation anxiety, social anxiety, and school fear in children with sleep bruxism, while other anxiety domains did not differ between groups [10]. However, findings are not uniform across studies, particularly when different methods are used to assess sleep bruxism [4]. 

Additionally, a mother's level of anxiety has been recognized as an important related factor, with her state anxiety frequently being associated with the severity of her child's bruxism [7,10]. Many anxiety assessment tools provide global anxiety scores and were originally developed for adults, potentially limiting their suitability for children [12]. The Spence Children’s Anxiety Scale - Parent Version (SCAS-P), on the other hand, is a validated tool that measures symptoms across six anxiety domains corresponding to DSM-IV categories, providing a multidimensional and age-appropriate analysis of anxiety [13]. Its key benefit is its capacity to identify specific anxiety domains (such as separation anxiety, social phobia, and obsessive-compulsive symptoms), thus providing a more comprehensive clinical picture than other anxiety surveys [14,15]. Although multidimensional evidence is available, it remains limited and has varied according to the anxiety instrument and the method used to assess sleep bruxism. Therefore, the present study aimed to further characterize overall and domain-specific anxiety in children with and without sleep bruxism using the multidimensional Spence Children’s Anxiety Scale - Parent Version (SCAS-P).

## Materials and Methods

*Study design*

An observational, analytical, cross-sectional study with a quantitative approach was conducted to evaluate the association between anxiety levels and sleep bruxism in pediatric patients.

*Setting*

The study was conducted at the dental clinic of Universidad Andrés Bello (Viña del Mar, Chile).

*Participants*

Pediatric patients were included in the study if they met the following criteria: were aged 7-12 years and receiving dental care at the university clinic; had an available legal guardian living with the child; had written informed consent provided by the guardian; provided positive assent; and had complete responses to both the sleep bruxism and anxiety questionnaires. Subjects were excluded if the guardian was unable to complete the questionnaires accurately, or if the clinical examination could not be completed due to lack of participant cooperation. Participants were selected using non-probabilistic convenience sampling. 

*Variables, data sources, and measurements*

Sleep bruxism, classified as present or absent, was assessed according to the diagnostic criteria of the International Classification of Sleep Disorders, 3rd edition (ICSD-3) based on reports of regular or frequent tooth-grinding sounds during sleep together with one or more of the following clinical signs and/or symptoms consistent with reports of tooth grinding during sleep: Abnormal tooth wear consistent with reported tooth grinding during sleep; transient morning jaw muscle pain or fatigue; temporal headache; jaw locking upon awakening consistent with reported tooth grinding during sleep [16]. Anxiety was assessed using the Spence Children’s Anxiety Scale - Parent Version (SCAS-P), which is a standardized and validated psychometric instrument for assessing anxiety symptoms in children based on parent reports. The questionnaire included 38 scored items rated on a 4-point Likert-like scale (from 0: Never to 3: Always), with a maximum total score of 114, where higher scores indicate greater anxiety severity [17]. In the present study, anxiety was operationalized using the SCAS-P total score. In addition, scores from each SCAS-P subscale were analyzed to explore specific anxiety domains, including Panic/Agoraphobia, Separation Anxiety, Physical Injury Fears, Social Phobia, Obsessive-Compulsive Symptoms, and Generalized Anxiety. Covariates included age (years), age group (7-9 years or 10-12 years), and sex (female/male).

*Bias*

To reduce measurement bias, examiners were calibrated prior to data collection, and standardized diagnostic criteria and validated instruments were used. Only questionnaires completed by caregivers living with the child were included.

*Study size*

An a priori sample size calculation, using G*Power (version 3.1), indicated that approximately 124 participants were required (Cohen’s d=0.50; alpha level of 0.05; power of 80%). The final sample included 122 participants.

*Statistical methods*

Statistical analyses were performed using JASP (version 0.19.3). Normality of continuous variables was assessed using the Shapiro-Wilk test. As anxiety-related variables and age showed considerable deviations from normality, group comparisons between children with sleep bruxism (SB) and without sleep bruxism (No-SB) were performed using the Mann-Whitney U test. Effect sizes for non-parametric comparisons were reported as rank-biserial correlations (r); values of 0.10, 0.30, and 0.50 were interpreted as small, moderate, and large effects, respectively. Associations between sleep bruxism and categorical variables (sex and age group) were evaluated using the chi-square test of independence. Effect sizes for chi-square analyses were estimated using the phi coefficient (φ). All statistical tests were two-tailed, and a *p* value <0.05 was considered statistically significant.

*Ethical considerations*

Written informed consent was obtained from legal guardians, and assent was obtained from all children prior to participation. The study protocol, design, and implementation were approved by the Scientific Ethics Committee of the Faculty of Dentistry of Andrés Bello University, Viña del Mar, Chile (Folder No. 2024_PP_020_B).

## Results

*Participant characteristics*

A total of 163 children were initially assessed for eligibility. Of these, 41 were excluded for failing to meet the inclusion criteria. Finally, the sample comprised 122 children included in the analysis. Eighty-eight children were identified as having sleep bruxism (SB), while 34 were classified as not having sleep bruxism (No-SB). The mean age was 9.4 ± 1.6 years in the No-SB subjects and 8.8 ± 1.3 years in SB subjects.

*Association between sleep bruxism and anxiety (SCAS-P total score)*

Children with SB showed significantly higher total anxiety scores on the SCAS-P compared with children without SB (median 27.0 vs. 21.0). The Mann-Whitney U test indicated a statistically significant difference between groups (U=961.5, *p*=0.002), with a moderate effect size (r=0.36). Figure 1 shows a rightward shift of SCAS-P total scores in children with SB compared with children without SB, indicating higher anxiety levels in the SB group. Although some overlap between distributions is observed, children with SB show higher medians and greater score dispersion. This visual pattern is consistent with the significant Mann-Whitney U test and the moderate effect size observed.

*Association between sleep bruxism and SCAS-P subscales*

Descriptive statistics of SCAS-P subscales are shown in Table 1. Significant group differences were observed in several anxiety subdomains. Compared with the No-SB group, children with SB showed higher scores for Panic/Agoraphobia (U=1078.5, *p*=0.014; r=0.28); Separation Anxiety (U=988.5, *p*=0.004; r=0.34); Physical Injury Fears (U=1012.0, *p*=0.005; r=0.32); and Social Phobia (U=1143.5, *p*=0.043; r=0.24). All effect sizes ranged from small to moderate. No statistically significant differences were found between groups for Obsessive-Compulsive Symptoms (U=1337.5, *p*=0.357; r=0.11) or Generalized Anxiety (U=1225.0, *p*=0.120; r=0.18).

*Age and sex*

Age did not differ significantly between groups (U=1831.0, *p*=0.051), although children with sleep bruxism tended to be younger. No association was found between sleep bruxism and sex (χ²=0.34, *p*=0.560).

*Age group and sleep bruxism*

A significant association was observed between sleep bruxism and age group (χ²=6.05, *p*=0.014). Sleep bruxism was more frequent in children aged 7-9 years compared with those aged 10-12 years. The effect size was small to moderate (φ=-0.22).

## Discussion

The results of the present cross-sectional study show that children with sleep bruxism have significantly higher overall anxiety levels compared with those without the condition, which aligns with prior research demonstrating a relationship between sleep bruxism and anxiety symptoms in children [6,10]. Nevertheless, effect sizes ranged from small to moderate, suggesting that while anxiety is an important factor linked to sleep bruxism, it is not the only one, highlighting the importance of focused psychological assessments rather than assuming the presence of generalized anxiety disorders. Previous studies have also explored domain-specific anxiety in children with sleep bruxism. In particular, Yazıcıoğlu and Ray [10], using the multidimensional SCARED instrument, reported higher total anxiety as well as higher separation anxiety, social anxiety, and school fear in children with sleep bruxism, whereas somatic/panic and generalized anxiety did not significantly differ between groups. Thus, the incremental contribution of the present study lies not in establishing a general association between anxiety and sleep bruxism, but in further characterizing its domain-specific pattern using a different multidimensional instrument and reporting the magnitude of these associations through effect-size estimates. The present findings partially replicate and extend this pattern using the SCAS-P. Separation Anxiety and Social Phobia were again significantly higher in children with sleep bruxism, while Generalized Anxiety remained non-significant. In addition, the present study identified higher Panic/Agoraphobia and Physical Injury Fears scores, whereas Obsessive-Compulsive Symptoms did not differ significantly between groups. 

However, the association between anxiety and sleep bruxism has not been consistently demonstrated across studies. Alfano *et al*. [4], using polysomnography to identify sleep bruxism, found no significant difference in sleep bruxism rates between children with generalized anxiety disorder and healthy controls and reported poor concordance between parent-reported and PSG-detected sleep bruxism. More recently, Büyüknalbant *et al*. [18] found no statistically significant overall association between anxiety and sleep bruxism in a sample recruited from a child and adolescent psychiatry clinic, although higher anxiety levels were observed among adolescents with sleep bruxism. These contrasting findings suggest that differences in sample characteristics, anxiety constructs, and sleep bruxism assessment methods may contribute to the heterogeneity of the available evidence. Accordingly, sleep bruxism is multifactorial, with systematic reviews suggesting that factors related to sleep and arousal mechanisms are more significant in its development than psychosocial variables alone [7,11,19].

The association observed between sleep bruxism and  the younger age group aligns with epidemiological studies showing that sleep bruxism is more common in early childhood and tends to decline as children get older [7]. This trend with age is thought to result from neurodevelopmental changes, the maturation of sleep patterns, and evolving emotional regulation throughout childhood [2]. In contrast, the absence of a relationship between sleep bruxism and sex in the present study is consistent with earlier research, which also indicates that its prevalence is comparable between boys and girls in pediatric populations [6,8].

Measuring anxiety using the SCAS-P represents a key strength of the present study. This instrument is specifically designed to capture a broad range of anxiety symptoms experienced by children, encompassing six domains: Panic/agoraphobia, separation anxiety, social phobia, physical injury fears, obsessive-compulsive symptoms, and generalized anxiety [13,17]. Validation studies involving Spanish-speaking children have demonstrated adequate reliability and construct validity, supporting its application in both clinical and research contexts [13,15]. Unlike shorter tools such as the GAD-7, which focuses mainly on generalized anxiety and is designed for adults, the SCAS-P offers a more comprehensive and age-appropriate assessment for children [15]. This comprehensive scope enabled the identification of associations between sleep bruxism and specific anxiety domains rather than generalized anxiety alone.

Parental reports of child health outcomes may introduce reporting bias, especially for internalizing symptoms such as anxiety, which may not always be directly observable by caregivers. Parents may either underrecognize emotional distress or overestimate symptoms influenced by their own psychological state or concern for the child’s well-being. This limitation has been acknowledged in studies assessing psychosocial factors and sleep bruxism in pediatric populations [4,20,21]. However, the SCAS-P was specifically developed as a parent-report tool and has shown sufficient validity and reliability for assessing anxiety-related behaviors in children, particularly in age groups where self-reports may not be reliable [13].

Certain limitations must be considered when interpreting these results. First, the cross-sectional design does not allow causal inferences regarding the relationship between anxiety and sleep bruxism, a limitation shared with most pediatric sleep bruxism studies [6,8]. Second, although instrumental confirmation was not performed, standardized ICSD-3 criteria were used, which are commonly adopted in epidemiological studies of pediatric sleep bruxism, and were considered appropriate for the research question [1]. Nevertheless, the absence of instrumental confirmation may have resulted in some degree of diagnostic misclassification. This limitation is particularly relevant because PSG-based assessment has not consistently reproduced associations observed in studies relying on clinical or report-based sleep bruxism assessment [4]. Third, participants were recruited using a non-probabilistic convenience sampling strategy from a single university dental clinic, which may limit the generalizability of the findings to other pediatric populations or clinical settings. Fourth, anxiety assessment relied exclusively on parental report, which may be influenced by reporting bias; however, parent-report instruments remain appropriate and are widely used in younger pediatric populations [13,15]. Fifth, although the final sample (n=122) was marginally below the a priori calculated sample size (n=124), the difference was minimal (1.6%) and is unlikely to have materially affected the statistical precision of the analyses. Finally, future longitudinal studies including multimodal anxiety assessment and instrumental sleep measures are warranted to clarify causal pathways and to further refine diagnostic and management strategies for pediatric sleep bruxism.

## Conclusions

Sleep bruxism in children was associated with higher levels of anxiety, particularly across specific anxiety domains. These findings highlight the relevance of psychosocial assessment in pediatric patients with sleep bruxism. 

## Figures and Tables

**Table 1:** Table 1: SCAS-P total and subscale scores according to sleep bruxism status. Values are presented as median (interquartile range) and mean ± standard deviation.

	**No-SB subjects (n=34)**	**SB subjects (n=88)**
**SCAS-P total score**	**21 (15.25); 21.75 ± 11.11**	**27 (21.5); 30.27 ± 14.20**
Panic/Agoraphobia	1 (2); 1.63 ± 1.96	2.5 (4); 3.24 ± 3.83
Separation Anxiety	5 (5); 5.43 ± 3.49	7 (4.75); 7.27 ± 3.05
Physical Injury Fears	3 (3); 3.56 ± 2.03	5 (3.75); 5.06 ± 2.86
Social Phobia	4 (4); 4.64 ± 3.41	5.5 (6); 6.29 ± 4.06
Obsessive-Compulsive Symptoms	1 (3); 1.92 ± 1.96	2 (4.75); 2.85 ± 3.11
Generalized Anxiety	4 (3); 4.59 ± 2.59	5 (3.5); 5.53 ± 2.96

SCAS-P: Spence Children’s Anxiety Scale - Parent Version; SB: Sleep bruxism.

**Figure 1:**
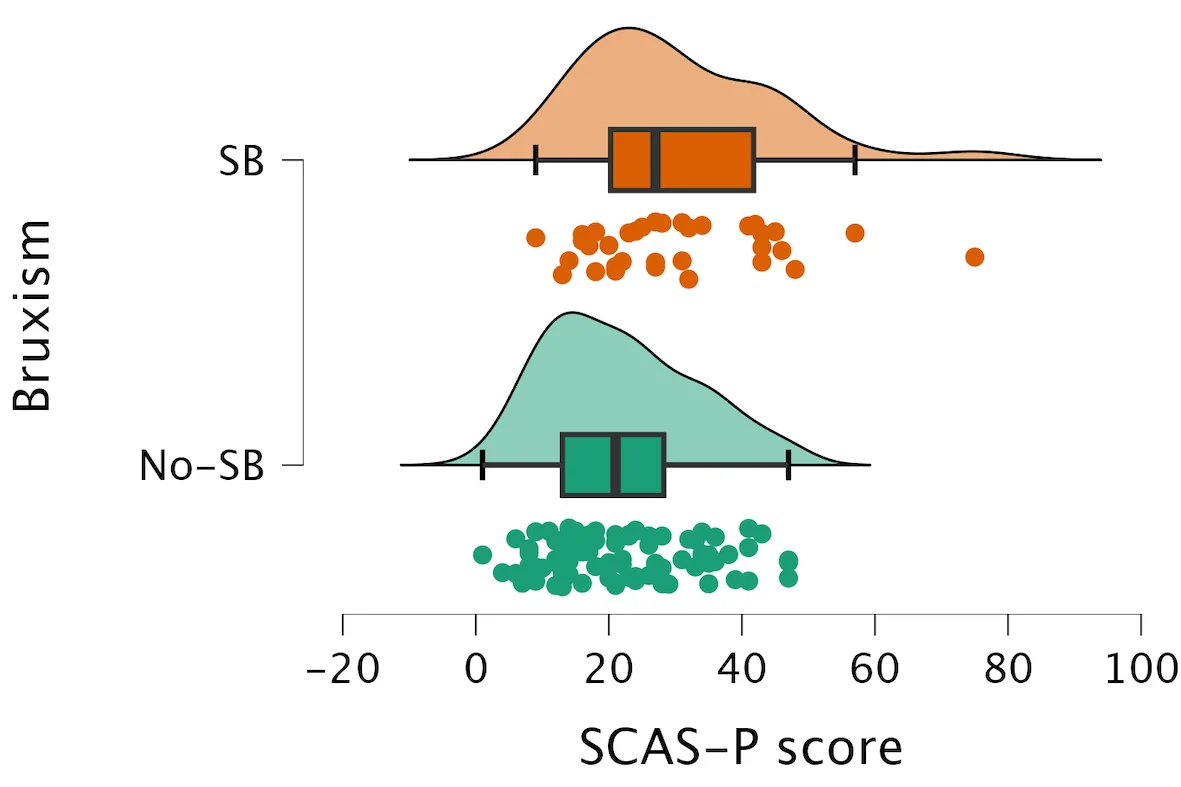
Fig. 1: Raincloud plot showing the distribution of total SCAS-P scores in children with sleep bruxism (SB) and without sleep bruxism (No-SB). Density plots represent score distributions, individual dots correspond to individual observations, and embedded boxplots display the median and interquartile range. Children with sleep bruxism exhibited higher SCAS-P scores than children without sleep bruxism.

## Data Availability

Declared none.
